# Modes of mechanical ventilation vary between hospitals and intensive care units within a university healthcare system: a retrospective observational study

**DOI:** 10.1186/s13104-018-3534-z

**Published:** 2018-07-03

**Authors:** Craig S. Jabaley, Robert F. Groff, Milad Sharifpour, Jayashree K. Raikhelkar, James M. Blum

**Affiliations:** 1 Division of Critical Care Medicine, Department of Anesthesiology, Emory University, 1364 Clifton Road NE, Atlanta, GA 30322 USA; 2 Division of Critical Care Medicine, Anesthesiology Service Line, Atlanta Veterans Affairs Medical Center, Decatur, GA USA; 3 Department of Biomedical Informatics, Emory University School of Medicine, Atlanta, GA USA

**Keywords:** Positive pressure ventilation, Respiratory failure, Mechanical ventilators, Noninvasive ventilation, Ventilator weaning, Intensive care

## Abstract

**Objective:**

As evidence-based guidance to aid clinicians with mechanical ventilation mode selection is scant, we sought to characterize the epidemiology thereof within a university healthcare system and hypothesized that nonconforming approaches could be readily identified. We conducted an exploratory retrospective observational database study of routinely recorded mechanical ventilation parameters between January 1, 2010 and December 31, 2016 from 12 intensive care units. Mode epoch count proportions were examined using Chi squared and Fisher exact tests as appropriate on an inter-unit basis with outlier detection for two test cases via post hoc pairwise analyses of a binomial regression model.

**Results:**

Final analysis included 559,734 mode epoch values. Significant heterogeneity was demonstrated between individual units (P < 0.05 for all comparisons). One unit demonstrated heightened utilization of high-frequency oscillatory ventilation, and three units demonstrated frequent synchronized intermittent mandatory ventilation utilization. Assist control ventilation was the most commonly recorded mode (51%), followed by adaptive support ventilation (23.1%). Volume-controlled modes were about twice as common as pressure-controlled modes (64.4% versus 35.6%). Our methodology provides a means by which to characterize the epidemiology of mechanical ventilation approaches and identify nonconforming practices. The observed variability warrants further clinical study about contributors and the impact on relevant outcomes.

**Electronic supplementary material:**

The online version of this article (10.1186/s13104-018-3534-z) contains supplementary material, which is available to authorized users.

## Introduction

Relatively scant evidence exists to guide clinicians in their selection of a mechanical ventilation (MV) mode. International epidemiological studies have identified that tidal volume (Vt), positive end-expiratory pressure (PEEP), and other parameters are beginning to align with lung protective ventilation strategies [1–4]. These and other characterizations of MV mode selection are variable in their scope, often examining only certain modes or patient subsets, and a clear picture of practice patterns remains elusive [5–21].

Developing a means by which to assess variability in the approach to MV offers an opportunity to identify outlying or nonconforming practices for which educational, quality improvement, or other such interventions could be appropriate [22]. For example, routine use of high-frequency oscillatory ventilation (HFOV) and synchronized intermittent mandatory ventilation (SIMV) have been called into question. Two prospective studies of HFOV demonstrated largely equivalent outcomes in patients with acute respiratory distress syndrome, and SIMV has been associated with delayed separation from MV [23–28].

In light of multiple barriers to the consistent and evidence-based selection of a MV mode, we sought to characterize the epidemiology of MV mode selection within four hospitals affiliated with a university healthcare system. We hypothesized that this approach could identify outlying or nonconforming approaches to MV, specifically the provision of HFOV and SIMV as test cases. Furthermore, we aimed to characterize variability between ICUs that treat similar patient populations and identify, if present, any consistent patterns between types of ICUs and between individual ICUs across hospitals in support of subsequent hypothesis generation.

## Main text

### Methods

#### Study design

We conducted an exploratory retrospective observational database study of routinely collected MV parameters to examine mode utilization in 12 adult ICUs across four hospitals between January 1, 2011 and December 31, 2016. Condensed reporting herein follows the Strengthening the Reporting of Observational Studies in Epidemiology statement and the Reporting of Studies Conducted Using Observational Routinely Collected Health Data statements where applicable [29, 30]. Approval was granted by the Emory University Institutional Review Board (ID #IRB00095006) with a waiver of informed consent owing to its retrospective design.

#### Setting and source population

Twelve ICUs in four standalone hospitals affiliated with Emory Healthcare (Atlanta, GA USA) were studied, which comprised 191/237 critical care beds, or 80.6% of the system wide total. We categorized ICUs by their primary mission whereby a: (a) cardiothoracic surgical ICU (CTICU) treats patients after major heart, lung, and vascular surgery; (b) neuroscience ICU (NSICU) treats patients following cerebrovascular insults and intracranial procedures; (c) surgical ICU (SICU) treats postoperative patients not in one of the two prior categories, (d) medical ICU (MICU) treats critically ill adults not having recently undergone surgery, and (e) medical-surgical ICU (MSICU) treats a mixture of critically ill adults. The studied ICUs vary in their format and staffing (see Additional file 1: Table S1 for further details) [31, 32]. Ventilator management is the responsibility of the critical care physician, or the admitting physician in ICUs without critical care staffing. MV equipment has changed across the system during the study period with increasing availability of Hamilton (Hamilton Medical AG, Bonaduz, Switzerland) ventilators (e.g. Galileo, G3, and G5) and a gradual reduction in Puritan Bennett (Covidien LP, Boulder, CO, USA) models (e.g. 840) as a result of standardization efforts.

#### Data sources

MV parameters and settings, including mode, are routinely charted every 4 h in the electronic medical record (EMR), or immediately following a setting change, by respiratory therapists (RTs). EMR documentation is consolidated nightly into the Clinical Data Warehouse (CDW) via an extract, transform, and load process, which has been internally validated by Information Services. The CDW is indexed to support advanced analytics and is accessed via structured query language and MicroStrategy (MicroStrategy Inc., Washington, DC, USA) interfaces.

#### Structured query approach

Each instance of recorded MV parameters was considered a stand-alone epoch and extracted from the CDW on a per-epoch, per-ICU basis during the period of interest and then imported into MariaDB (MariaDB Corporation AB, Espoo, Finland). No database linking was required. MariaDB was used to aggregate the data and identify nonsensical (e.g. numerical) values, which were excluded from analysis. Data cleaning consisted of identification and removal of elements with typographical errors (e.g. letter transposition and misspelling), which were rare as the EMR relies heavily on pre-populated dropdown charting for MV mode. The two NSICUs in Hospital 1 were considered in aggregate for inter-unit comparisons as they are managed by the same critical care team. Reported epoch counts represent all those recorded during the period of interest.

#### Statistical analysis

Proportions of routinely recorded nominal categorical mode counts were generated and initially visualized in SAS JMP Pro version 13.1.0 (SAS Institute Inc., Cary, NC, USA). Final analysis was done with R version 3.4.4 (R Core Team, Vienna, Austria) in RStudio 1.1.453 (RStudio Inc, Boston, MA, USA). Pearson’s Chi square test for overall homogeneity was run prior to further comparisons. Sub-analyses of nominal categorical MV mode count proportions were conducted using Chi square or Fisher’s exact test as appropriate on an inter-ICU basis. Modes accounting for less than 2% of per-unit epochs were excluded from this analysis as they may be less clinically significant. Outlying proportions of HFOV and SIMV (both pressure- and volume-controlled variants) as test cases were initially identified through examination of standardized residuals. These were confirmed via binomial regression with post hoc comparison of least-squares means and Bonferroni corrected pairwise comparisons to examine inter-ICU variability. P values < 0.05 were considered significant. Given the limited dataset and goals of the study, characterization of demographic variables and attempts to control for bias or confounding were beyond the scope of the investigation. Figures were generated using the ggplot2 and RColorBrewer packages [33].

### Results

**Fig. 1:**
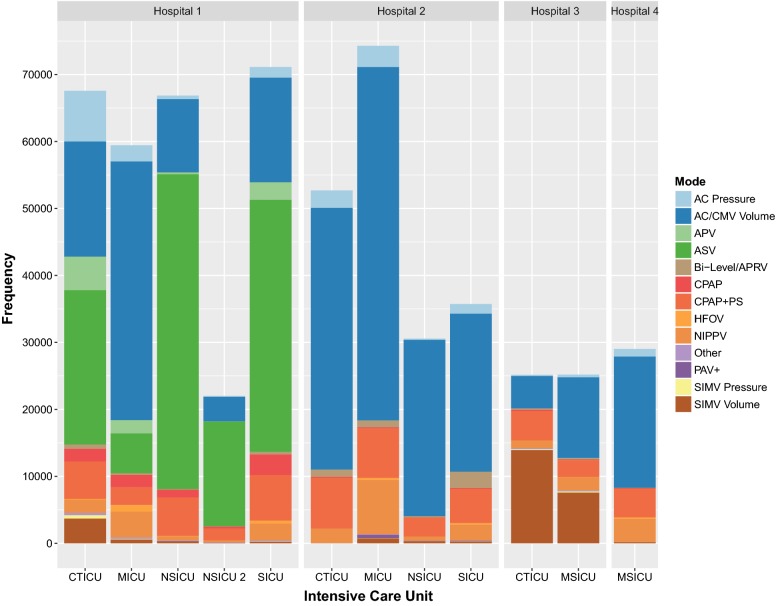
Mechanical ventilation mode epoch counts per intensive care unit by hospital. See list of abbreviations section

**Fig. 2:**
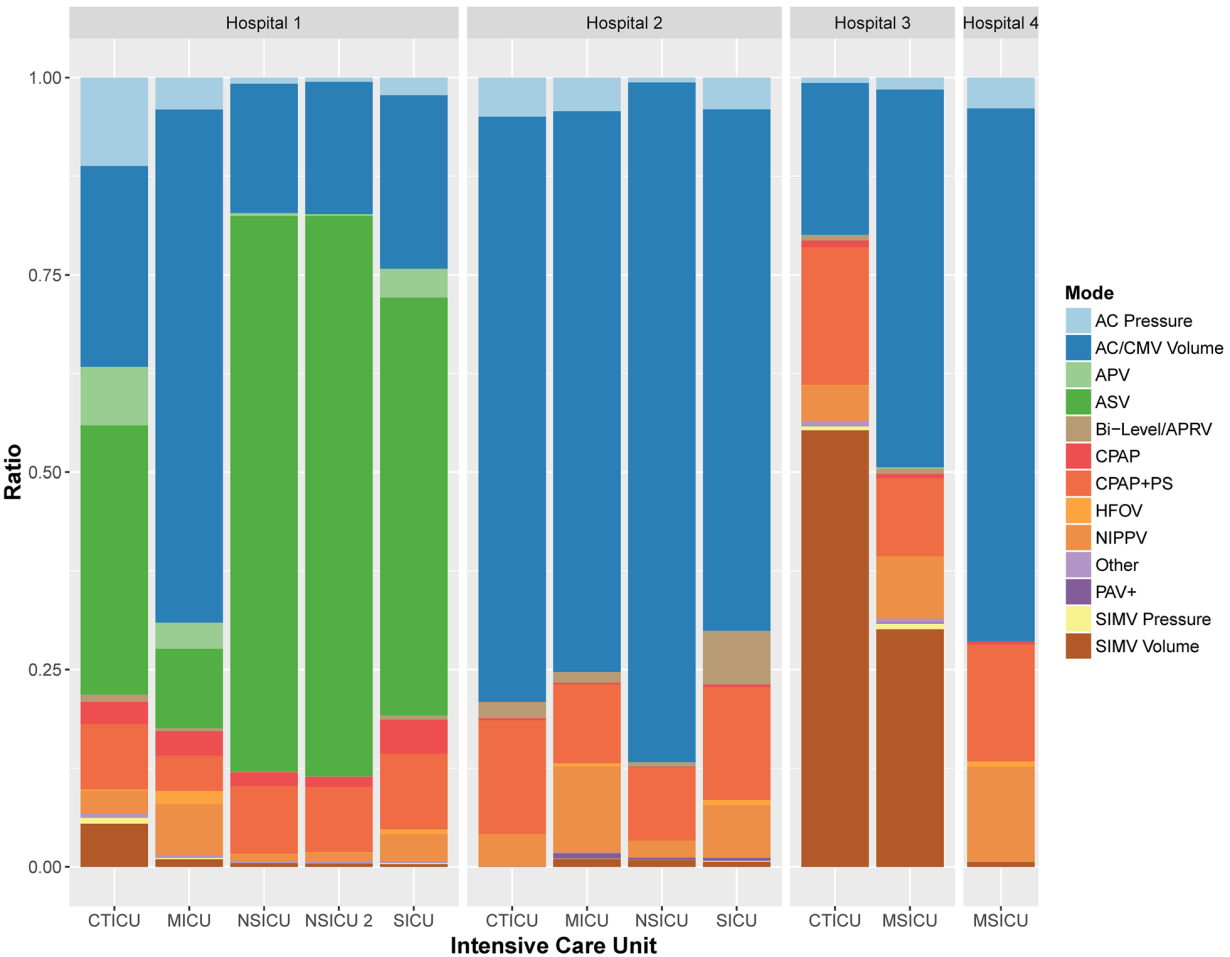
Ratios of mechanical ventilation mode epochs per intensive care unit by hospital. See list of abbreviations section

The CDW query identified 559,762 recorded MV epochs from the ICUs of interest between January 1, 2011 and December 31, 2016. Of those, the MV mode values for 28 epochs (0.005%) were nonsensical and excluded from analysis (Additional file 2: Figure S1). Mode epoch frequencies were significantly heterogeneous between individual ICUs as depicted with aggregate counts in Fig. 1 and proportions in Fig. 2 (Table 1, P < 0.05 for all comparisons). Heterogeneity was therefore evident between like types of ICU (Additional file 3: Table S2) as depicted proportionally on a per-unit basis in Additional file 4: Figure S2 and aggregated in Additional file 5: Figure S3. Similar variability was evident when aggregating units by hospital as depicted proportionally in Additional file 6: Figure S4.

**Table 1:** Aggregate mechanical ventilation mode epoch counts

Hospital	ICU	AC pressure	AC/CMV volume	APV	ASV	Bi-level/APRV	CPAP	CPAP + PS	HFOV	NIPPV	Other	PAV+	SIMV pressure	SIMV volume	All
		N (row %)	N (row %)	N (row %)	N (row %)	N (row %)	N (row %)	N (row %)	N (row %)	N (row %)	N (row %)	N (row %)	N (row %)	N (row %)	N
1	CTICU	7557 (11.2%)	17,196 (25.5%)	5011 (7.4%)	23,050 (34.1%)	628 (0.9%)	1891 (2.8%)	5593 (8.3%)	117 (0.2%)	1980 (2.9%)	340 (0.5%)	0 (0.0%)	510 (0.8%)	3699 (5.5%)	67,572
1	MICU	2401 (4.0%)	38,647 (65.0%)	1976 (3.3%)	5965 (10.0%)	221 (0.4%)	1850 (3.1%)	2660 (4.5%)	998 (1.7%)	3841 (6.5%)	214 (0.4%)	0 (0.0%)	92 (0.2%)	580 (1.0%)	59,445
1	NSICU	514 (0.8%)	10,957 (16.4%)	266 (0.4%)	47,034 (70.3%)	65 (0.1%)	1167 (1.8%)	5758 (8.6%)	77 (0.1%)	564 (0.8%)	149 (0.2%)	0 (0.0%)	3 (0.0%)	313 (0.5%)	66,867
1	NSICU 2	117 (0.5%)	3694 (16.8%)	46 (0.2%)	15,628 (71.0%)	18 (0.1%)	284 (1.3%)	1818 (8.3%)	17 (0.1%)	227 (1.0%)	75 (0.3%)	0 (0.0%)	1 (0.0%)	93 (0.4%)	22,018
1	SICU	1579 (2.2%)	15,631 (22.0%)	2606 (3.7%)	37,652 (52.9%)	374 (0.5%)	3066 (4.3%)	6828 (9.6%)	444 (0.6%)	2462 (3.5%)	149 (0.2%)	0 (0.0%)	77 (0.1%)	256 (0.4%)	71,124
2	CTICU	2609 (5.0%)	39,078 (74.1%)	0 (0.0%)	2 (0.0%)	1085 (2.1%)	122 (0.2%)	7607 (14.4%)	2 (0.0%)	2149 (4.1%)	9 (0.0%)	6 (0.0%)	14 (0.0%)	22 (0.0%)	52,705
2	MICU	3163 (4.3%)	52,784 (71.0%)	3 (0.0%)	2 (0.0%)	1015 (1.4%)	153 (0.2%)	7413 (10.0%)	303 (0.4%)	8109 (10.9%)	56 (0.1%)	522 (0.7%)	27 (0.0%)	749 (1.0%)	74,299
2	NSICU	183 (0.6%)	26,328 (86.1%)	2 (0.0%)	1 (0.0%)	173 (0.6%)	29 (0.1%)	2839 (9.3%)	0 (0.0%)	657 (2.2%)	2 (0.0%)	84 (0.3%)	2 (0.0%)	272 (0.9%)	30,572
2	SICU	1432 (4.0%)	23,617 (66.1%)	2 (0.0%)	0 (0.0%)	2444 (6.8%)	116 (0.3%)	5105 (14.3%)	240 (0.7%)	2367 (6.6%)	22 (0.1%)	123 (0.3%)	41 (0.1%)	240 (0.7%)	35,749
3	CTICU	169 (0.7%)	4848 (19.3%)	13 (0.1%)	1 (0.0%)	161 (0.6%)	211 (0.8%)	4402 (17.5%)	22 (0.1%)	1117 (4.4%)	172 (0.7%)	4 (0.0%)	128 (0.5%)	13,921 (55.3%)	25,169
3	MSICU	380 (1.5%)	12,061 (47.9%)	38 (0.2%)	3 (0.0%)	167 (0.7%)	130 (0.5%)	2505 (9.9%)	19 (0.1%)	1962 (7.8%)	129 (0.5%)	27 (0.1%)	183 (0.7%)	7586 (30.1%)	25,190
4	MSICU	1131 (3.9%)	19,593 (67.5%)	3 (0.0%)	3 (0.0%)	5 (0.0%)	110 (0.4%)	4294 (14.8%)	204 (0.7%)	3472 (12.0%)	17 (0.1%)	0 (0.0%)	7 (0.0%)	185 (0.6%)	29,024
All	All	21,235 (3.8%)	264,434 (47.2%)	9966 (1.8%)	129,341 (23.1%)	6356 (1.1%)	9129 (1.6%)	56,822 (10.2%)	2443 (0.4%)	28,907 (5.2%)	1334 (0.2%)	766 (0.1%)	1085 (0.2%)	27,916 (5.0%)	559,734

See list of abbreviations section

HFOV utilization was found to be nonconforming in single MICU in Hospital 1, which accounted for 40.9% of system wide epochs (P < 0.001 for all pairwise comparisons). Three ICUs similarly accounted for a disproportionate proportion of overall SIMV utilization: Hospital 3 CTICU with 48.4% of epochs (N = 14,049/29,001), Hospital 3 MSICU with 26.8% (N = 7769/29,001), and Hospital 1 CTICU with 14.5% (N = 4209/29,001; adjusted P < 0.001 for all pairwise comparisons).

Hospital 1 accounted for 51.3% of total recorded MV mode epochs (N = 287,026/559,734). Assist control (AC) was the most commonly recorded MV mode overall (N = 285,669/559,734, 51%) followed by adaptive support ventilation (ASV, N = 129,341/559,734, 23.1%), and pressure support ventilation (PSV, i.e. continuous positive airway pressure with pressure support [CPAP with PS], N = 56,822/559,734, 10.2%). When examining AC, SIMV, adaptive pressure ventilation (APV), and considering ASV to be a pressure-controlled mode for passive patients, the overall prevalence of invasive pressure controlled modes of ventilation was 35.6% (N = 161,627/453,977) compared to 64.4% for volume controlled modes (N = 292,350/453,977). In examining graphical comparisons, the two studied MICUs demonstrated the most consistent approach to mode selection when examining like-type ICUs (Additional file 4: Figure S2.) Within individual hospitals with greater than one ICU, units in Hospital 2 were the most consistent (Fig. 2).

### Discussion

Mode epoch proportions were significantly heterogeneous between individual ICUs, contributing to variability on an aggregate basis. Outlying utilization of HFOV in one ICU and SIMV in three suggests that this approach can be utilized to identify potentially nonconforming practice patterns. Only one hospital demonstrated a relatively consistent distribution of MV modes across its studied ICUs, and the two studied MICUs were most similar to one another; however, there were still statistically significant differences.

Nonconforming MV mode utilization was readily identified, and our approach may be able, more broadly, to identify other clinically outlying or inappropriate MV parameter selections. Utilization of HFOV appeared to be rare, which aligns with findings from large prospective studies suggesting equivalent clinical outcomes with greater risks than more conventional forms of MV [23, 24]. Overall utilization of SIMV was lower than the 26% rate reported in a recent large observational study, although we were able to identify outlying units [3]. Its routine application as a weaning modality has been questioned, and global utilization may be experiencing an associated decline [25–28, 34].

In keeping with prior studies demonstrating an approximate utilization rate of 50–60%, AC was likewise found to be the most frequently recorded mode of ventilation in our healthcare system [5, 6, 13, 35]. Recent international MV practice studies have, however, suggested that novel modes may be gradually driving a move away from AC toward closed-loop approaches [3]. Volume-controlled ventilation was about twice as common as pressure-controlled ventilation, which is in contrast to a recent international epidemiological study demonstrating the opposite [3]. From a speculative standpoint, this could reflect an emphasis on monitoring of Vt, greater ease of Vt restriction with volume-controlled modes, mischarting of volume-targeted pressure-controlled modes, or physician preference as no definitive difference in compliance, gas exchange, or outcomes has been demonstrated [36].

As discussed subsequently, limited inferences can be drawn about weaning approaches. Although one hospital demonstrated marked adoption of ASV, proportional assist ventilation (PAV) was very uncommon, which may suggest heterogeneity in the willingness to adopt closed-loop MV modes consistent with the findings of other epidemiological studies [3]. PSV was utilized relatively consistently across the system and in excess of CPAP alone, which is consistent with current MV weaning guidelines [37].

## Limitations

Our study has important limitations. Owing to its retrospective nature and use of a limited dataset, both unmeasured confounding with associated confounding bias and indication bias preclude inferences as to the etiology of heterogeneity, which may have been clinically appropriate. Rather, posited causes of heterogeneity herein are purely speculative. Staffing, equipment, patient-specific considerations, or any number of other factors may influence MV mode selection. Attempts to control for bias or confounding were beyond the scope of our epidemiological study, and these considerations limit generalizability of the findings.

Owing to our methodologic approach, we were unable to determine the duration or time sequence of specific MV modes utilization. As a simple example, although MV charting is consistent every 4 h or with changes, a patient on AC for 3 h and PSV for one would have a single AC and PSV epoch recorded. As such, the actual AC:PSV time ratio of 3:1 would appear to be 1:1 via this study’s methodology. This limits the inferences that can be drawn about approaches to ventilator separation and weaning.

As with any study that relies on routinely collected data, inaccurate bedside charting cannot be excluded. The incidence of nonsensical MV mode values was very low, likely owing to the EMR’s use of predefined MV mode selections. In that sense, our nominal categorical data may be more accurate than similarly recorded ordinal data. However, the potential for information bias cannot be excluded. For example, pressure-controlled volume-targeted AC modes could conceivably be charted incorrectly.

The highly heterogeneous nature of MV mode distribution found in the current study suggests that mode selection likely involves a complex interplay of factors, which could include institution, ICU, or provider-specific considerations in addition to patient or disease-related factors. The relationship between MV mode selection, MV parameters (e.g. Vt, PEEP, plateau pressure, or driving pressure), and clinical outcomes also warrants further investigation, especially as approaches to mode selection appear highly variable.

## Additional files

**Additional file 1: Table S1.** Details of studied hospitals and intensive care units.

**Additional file 2: Figure S1.** CONSORT flow diagram.

**Additional file 3: Table S2.** Mechanical ventilation mode epochs per intensive care unit type.

**Additional file 4: Figure S2.** Ratios of mechanical ventilation mode epochs per hospital by intensive care unit type. Hospital 1 NSICUs depicted in aggregate. See list of abbreviations section.

**Additional file 5: Figure S3.** Ratios of mechanical ventilation mode epochs per intensive care unit type. See list of abbreviations section.

**Additional file 6: Figure S4.** Ratios of mechanical ventilation mode epochs per hospital. See list of abbreviations section.

## Abbreviations

AC: assist control
APRV: airway pressure release ventilation
APV: adaptive pressure ventilation
ASV: adaptive support ventilation
CDW: Clinical Data Warehouse
CMV: continuous mandatory ventilation
CPAP: continuous positive airway pressure
CPAP + PS: continuous positive airway pressure with pressure support (i.e. pressure support ventilation [PSV])
CTICU: cardiothoracic intensive care unit
EMR: electronic medical record
HFOV: high frequency oscillatory ventilation
ICU: intensive care unit
MICU: medical intensive care unit
MSICU: medical-surgical intensive care unit
MV: mechanical ventilation
NIPPV: non-invasive positive pressure ventilation
NSICU: neuroscience intensive care unit
PAV: proportional assist ventilation
PEEP: positive end-expiratory pressure
Pplat: plateau pressure
PS: pressure support
PSV: pressure support ventilation
RT: respiratory therapist
SICU: surgical intensive care unit
SIMV: synchronized intermittent mandatory ventilation
