# Supplementary material for: Modes of mechanical ventilation vary between hospitals and intensive care units within a university healthcare system: a retrospective observational study
Source: BMC Res Notes. 2018 Jul 3;11:425. doi: 10.1186/s13104-018-3534-z (PMC6029057; doi:10.1186/s13104-018-3534-z)
Supplement: Supplementary file 1 — Additional file 1: Table S1. Details of studied hospitals and intensive care units. [file 13104_2018_3534_MOESM1_ESM.docx]

**Table S1 Title: Details of Studied Hospitals and Intensive Care Units**

| **Hospital** | **Practice Type** | **Referral Center** | **ICU** | **Format** | **Critical Care Physician Staffing** | **Primary Physician Staffing Specialty** | **Critical Care Trainees** |
| --- | --- | --- | --- | --- | --- | --- | --- |
| 1 | Academic | Quaternary | CTICU | Hybrid | Yes | Anesthesiology* | Yes |
|  |  |  | MICU | Closed | Yes | Pulmonology | Yes |
|  |  |  | NSICU 1 & 2 | Hybrid | Yes | Neurology* | Yes |
|  |  |  | SICU | Hybrid | Yes | Surgery* | Yes |
| 2 | Hybrid | Tertiary | CTICU | Hybrid | Yes | Anesthesiology | No |
|  |  |  | MICU | Closed | Yes | Pulmonology | Yes |
|  |  |  | NSICU | Closed | Yes | Neurology | Variable |
|  |  |  | SICU | Hybrid | Yes | Pulmonology* | No |
| 3 | Hybrid | No | CTICU | Open | No | Surgery | No |
|  |  |  | MSICU | Hybrid | Yes | Pulmonology* | Variable |
| 4 | Community | No | MSICU | Open | No | Internal Medicine | No |

Table S1 Caption: *Denotes ICUs with a multidisciplinary staffing model. See list of abbreviations.
