# Supplementary material for: Modes of mechanical ventilation vary between hospitals and intensive care units within a university healthcare system: a retrospective observational study
Source: BMC Res Notes. 2018 Jul 3;11:425. doi: 10.1186/s13104-018-3534-z (PMC6029057; doi:10.1186/s13104-018-3534-z)
Supplement: Supplementary file 2 — Additional file 2: Figure S1. CONSORT flow diagram. [file 13104_2018_3534_MOESM2_ESM.pdf]

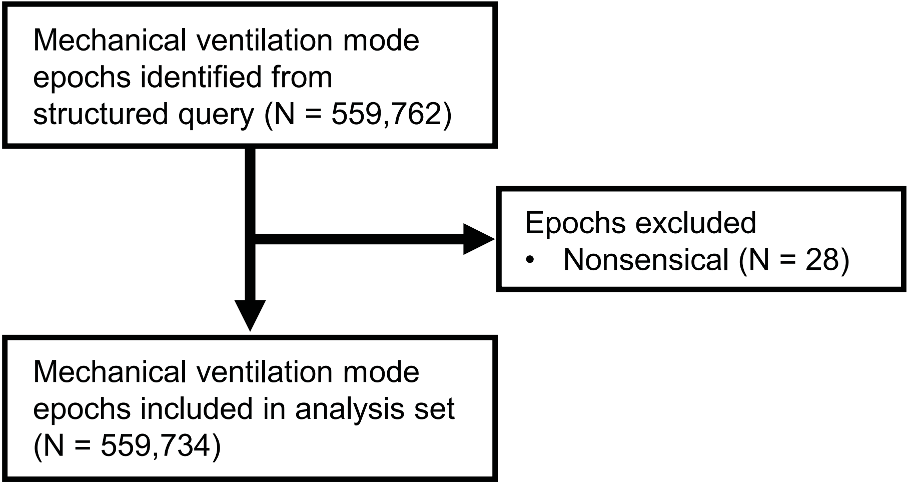
Mechanical ventilation mode epochs identified from structured query (N = 559,762)
Epochs excluded
• Nonsensical (N = 28)
Mechanical ventilation mode epochs included in analysis set (N = 559,734)
