# Supplementary material for: Modes of mechanical ventilation vary between hospitals and intensive care units within a university healthcare system: a retrospective observational study
Source: BMC Res Notes. 2018 Jul 3;11:425. doi: 10.1186/s13104-018-3534-z (PMC6029057; doi:10.1186/s13104-018-3534-z)
Supplement: Supplementary file 3 — Additional file 3: Table S2. Mechanical ventilation mode epochs per intensive care unit type. [file 13104_2018_3534_MOESM3_ESM.docx]

**Table S2 Title: Mechanical Ventilation Mode Epochs per Intensive Care Unit Type**

|  | Type of ICU |  |  |  |  |  |
| --- | --- | --- | --- | --- | --- | --- |
| Mode | CTICU N (Column %) | MICU N (Column %) | MSICU N (Column %) | NSICU N (Column %) | SICU N (Column %) | All  N (Column %) |
| AC Pressure | 10335 (7.11%) | 5564 (4.16%) | 1511 (2.79%) | 814 (0.68%) | 3011 (2.82%) | 21235 (3.79%) |
| AC/CMV Volume | 61122 (42.02%) | 91431 (68.36%) | 31654 (58.39%) | 40979 (34.3%) | 39248 (36.72%) | 264434 (47.24%) |
| APV | 5024 (3.45%) | 1979 (1.48%) | 41 (0.08%) | 314 (0.26%) | 2608 (2.44%) | 9966 (1.78%) |
| ASV | 23053 (15.85%) | 5967 (4.46%) | 6 (0.01%) | 62663 (52.46%) | 37652 (35.23%) | 129341 (23.11%) |
| Bi-Level/ APRV | 1874 (1.29%) | 1236 (0.92%) | 172 (0.32%) | 256 (0.21%) | 2818 (2.64%) | 6356 (1.14%) |
| CPAP | 2224 (1.53%) | 2003 (1.5%) | 240 (0.44%) | 1480 (1.24%) | 3182 (2.98%) | 9129 (1.63%) |
| CPAP+PS | 17602 (12.1%) | 10073 (7.53%) | 6799 (12.54%) | 10415 (8.72%) | 11933 (11.17%) | 56822 (10.15%) |
| HFOV | 141 (0.1%) | 1301 (0.97%) | 223 (0.41%) | 94 (0.08%) | 684 (0.64%) | 2443 (0.44%) |
| NIPPV | 5246 (3.61%) | 11950 (8.93%) | 5434 (10.02%) | 1448 (1.21%) | 4829 (4.52%) | 28907 (5.16%) |
| Other | 521 (0.36%) | 270 (0.2%) | 146 (0.27%) | 226 (0.19%) | 171 (0.16%) | 1334 (0.24%) |
| PAV+ | 10 (0.01%) | 522 (0.39%) | 27 (0.05%) | 84 (0.07%) | 123 (0.12%) | 766 (0.14%) |
| SIMV Pressure | 652 (0.45%) | 119 (0.09%) | 190 (0.35%) | 6 (0.01%) | 118 (0.11%) | 1085 (0.19%) |
| SIMV Volume | 17642 (12.13%) | 1329 (0.99%) | 7771 (14.33%) | 678 (0.57%) | 496 (0.46%) | 27916 (4.99%) |
| All (% total) | 145446 (26%) | 133744 (23.9%) | 54214 (9.7%) | 119457 (21.3%) | 106873 (19.1%) | 559734 |

Table S2 Caption: See list of abbreviations.
