# Supplementary figures and images for: Modes of mechanical ventilation vary between hospitals and intensive care units within a university healthcare system: a retrospective observational study
Source: BMC Res Notes. 2018 Jul 3;11:425. doi: 10.1186/s13104-018-3534-z (PMC6029057; doi:10.1186/s13104-018-3534-z)

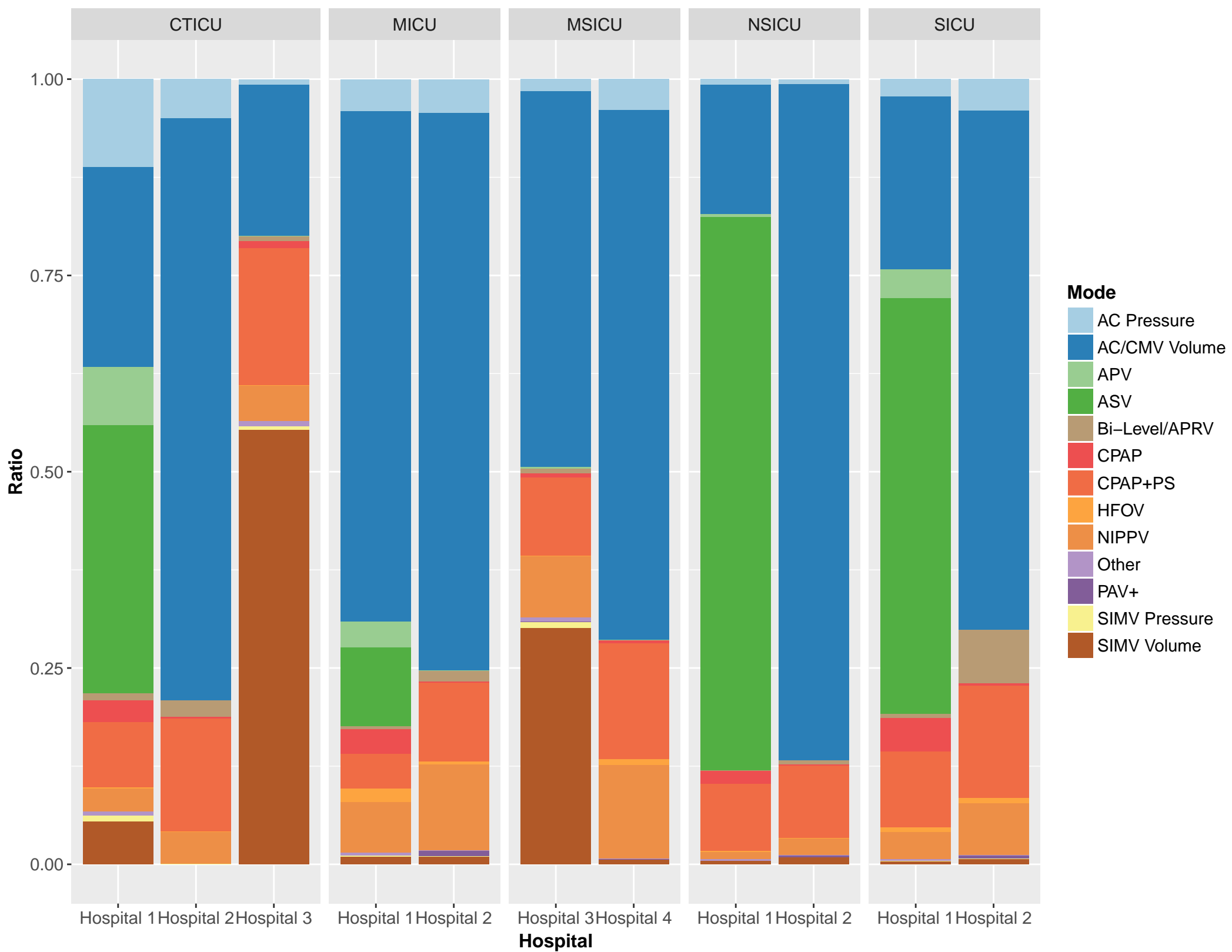

Supplement: Supplementary file 4 — Additional file 4: Figure S2. Ratios of mechanical ventilation mode epochs per hospital by intensive care unit type. Hospital 1 NSICUs depicted in aggregate. See list of abbreviations section. [file 13104_2018_3534_MOESM4_ESM.pdf]

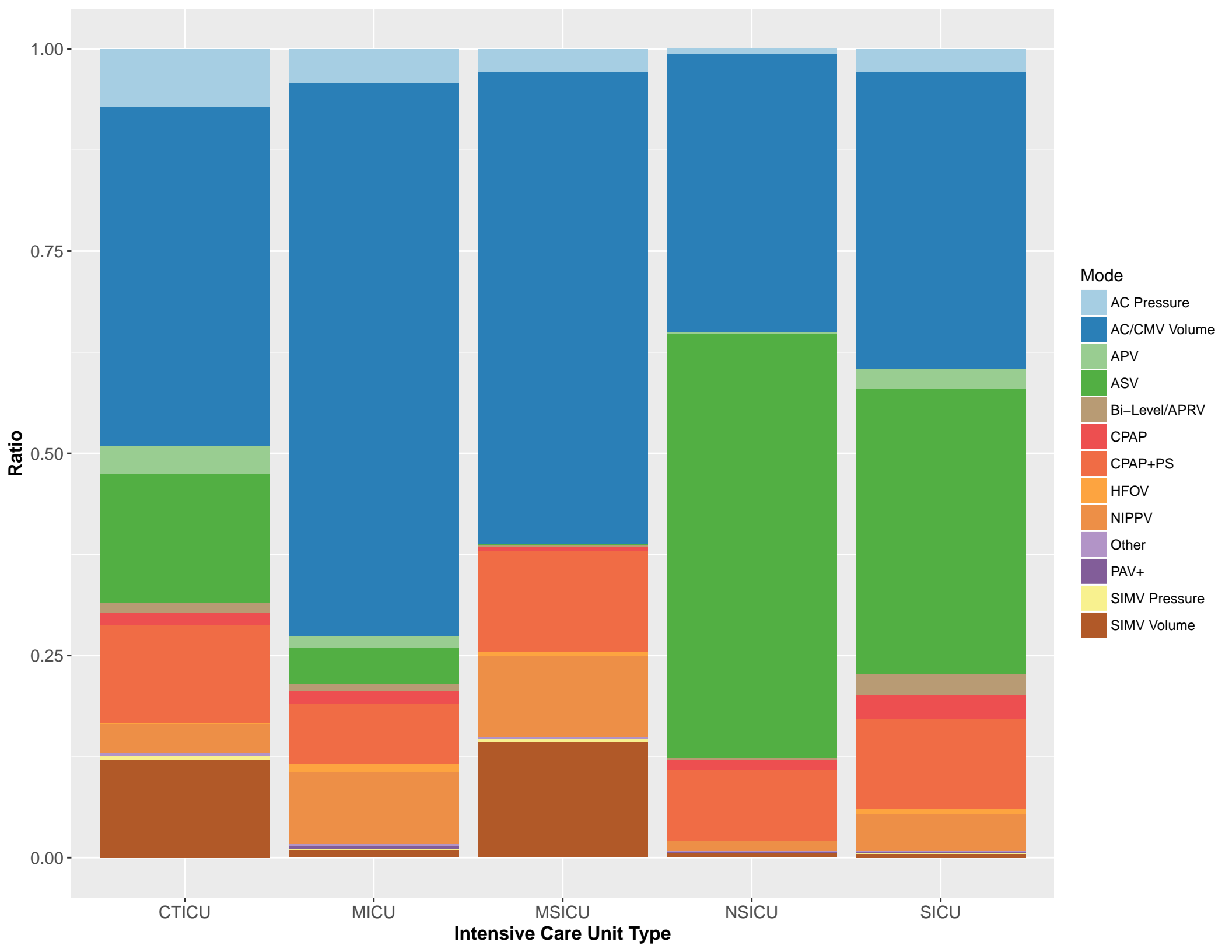

Supplement: Supplementary file 5 — Additional file 5: Figure S3. Ratios of mechanical ventilation mode epochs per intensive care unit type. See list of abbreviations section. [file 13104_2018_3534_MOESM5_ESM.pdf]

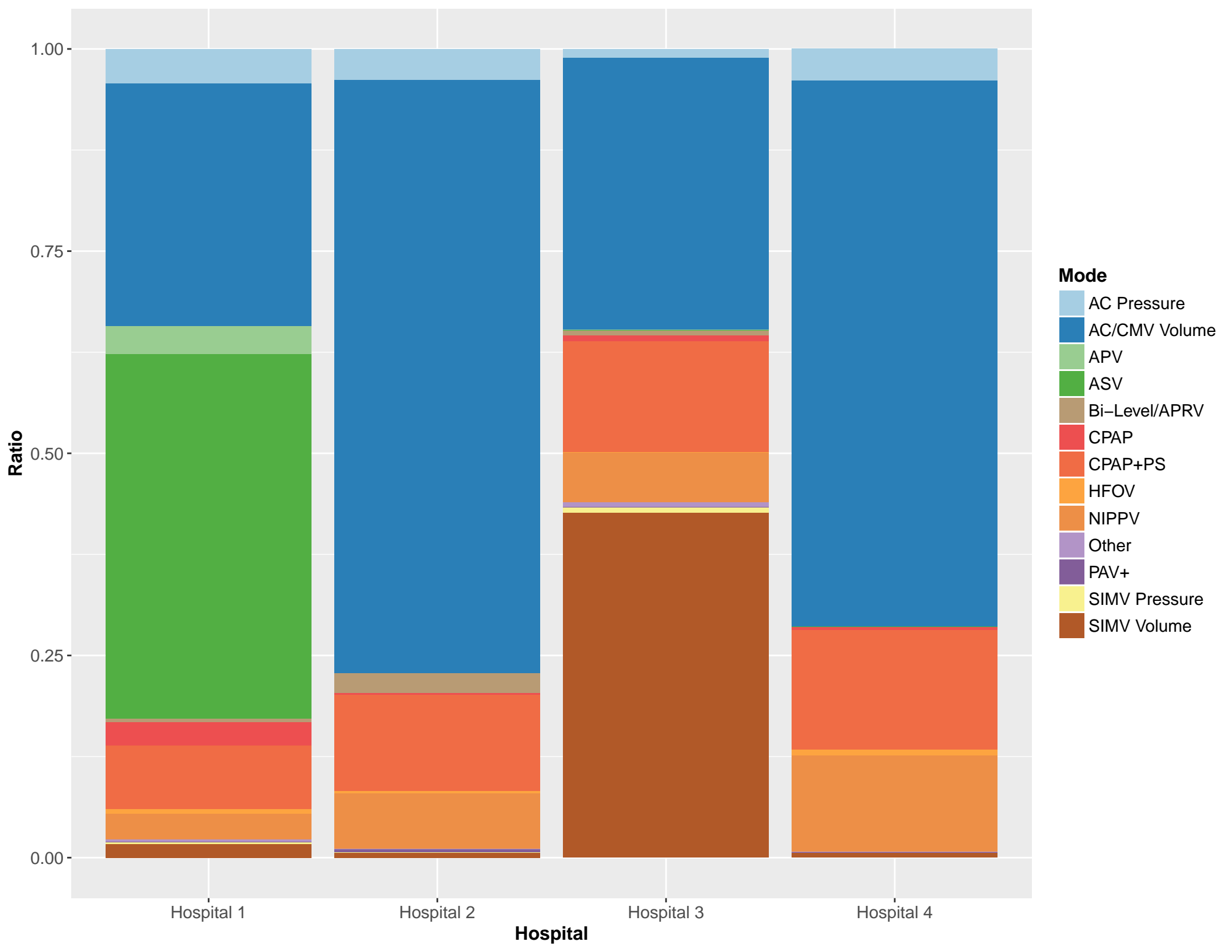

Supplement: Supplementary file 6 — Additional file 6: Figure S4. Ratios of mechanical ventilation mode epochs per hospital. See list of abbreviations section. [file 13104_2018_3534_MOESM6_ESM.pdf]
